# Investigate the origins of COVID-19

**DOI:** 10.1126/science.abj0016

**Published:** 2021-05-14

**Authors:** Jesse D. Bloom, Yujia Alina Chan, Ralph S. Baric, Pamela J. Bjorkman, Sarah Cobey, Benjamin E. Deverman, David N. Fisman, Ravindra Gupta, Akiko Iwasaki, Marc Lipsitch, Ruslan Medzhitov, Richard A. Neher, Rasmus Nielsen, Nick Patterson, Tim Stearns, Erik van Nimwegen, Michael Worobey, David A. Relman

**Affiliations:** 1 Basic Sciences and Computational Biology, Fred Hutchinson Cancer Research Center, Seattle, WA 98109, USA.; 2 Howard Hughes Medical Institute, Chevy Chase, MD 20815, USA.; 3 Stanley Center for Psychiatric Research, Broad Institute of Massachusetts Institute of Technology and Harvard University, Cambridge, MA 02142, USA.; 4 Department of Epidemiology and Department of Microbiology & Immunology, University of North Carolina at Chapel Hill, Chapel Hill, NC 27599, USA.; 5 Division of Biology and Biological Engineering, California Institute of Technology, Pasadena, CA 91125, USA.; 6 Department of Ecology and Evolution, University of Chicago, Chicago, IL 60637, USA.; 7 Dalla Lana School of Public Health, University of Toronto, Toronto, ON M5S 1A8, Canada.; 8 Cambridge Institute of Therapeutic Immunology & Infectious Disease, Cambridge, UK.; 9 Department of Immunobiology, Yale University School of Medicine, New Haven, CT 06519, USA.; 10 Department of Immunology and Infectious Diseases and Center for Communicable Disease Dynamics, Department of Epidemiology, Harvard T. H. Chan School of Public Health, Boston, MA 02115, USA.; 11 Biozentrum, University of Basel and Swiss Institute of Bioinformatics, Basel, Switzerland.; 12 Department of Integrative Biology and Department of Statistics, University of California, Berkeley, CA 94720, USA.; 13 Department of Human Evolutionary Biology, Harvard University, Cambridge, MA 02138, USA.; 14 Department of Biology and Department of Genetics, Stanford University, Stanford, CA 94305, USA.; 15 Department of Ecology and Evolutionary Biology, University of Arizona, Tucson, AZ 85721, USA.; 16 Department of Medicine and Department of Microbiology & Immunology, Stanford University School of Medicine, Stanford, CA 94305, USA.; 17 Center for International Security and Cooperation, Stanford University, Stanford, CA 94305, USA.

On 30 December 2019, the Program for Monitoring Emerging Diseases notified the world about a pneumonia of unknown cause in Wuhan, China (1). Since then, scientists have made remarkable progress in understanding the causative agent, severe acute respiratory syndrome coronavirus 2 (SARS-CoV-2), its transmission, pathogenesis, and mitigation by vaccines, therapeutics, and non-pharmaceutical interventions. Yet more investigation is still needed to determine the origin of the pandemic. Theories of accidental release from a lab and zoonotic spillover both remain viable. Knowing how COVID-19 emerged is critical for informing global strategies to mitigate the risk of future outbreaks.

In May 2020, the World Health Assembly requested that the World Health Organization (WHO) director-general work closely with partners to determine the origins of SARS-CoV-2 (2). In November, the Terms of Reference for a China–WHO joint study were released (3). The information, data, and samples for the study’s first phase were collected and summarized by the Chinese half of the team; the rest of the team built on this analysis. Although there were no findings in clear support of either a natural spillover or a lab accident, the team assessed a zoonotic spillover from an intermediate host as “likely to very likely,” and a laboratory incident as “extremely unlikely” [(4), p. 9]. Furthermore, the two theories were not given balanced consideration. Only 4 of the 313 pages of the report and its annexes addressed the possibility of a laboratory accident (4). Notably, WHO Director-General Tedros Ghebreyesus commented that the report’s consideration of evidence supporting a laboratory accident was insufficient and offered to provide additional resources to fully evaluate the possibility (5).

As scientists with relevant expertise, we agree with the WHO director-general (5), the United States and 13 other countries (6), and the European Union (7) that greater clarity about the origins of this pandemic is necessary and feasible to achieve. We must take hypotheses about both natural and laboratory spillovers seriously until we have sufficient data. A proper investigation should be transparent, objective, data-driven, inclusive of broad expertise, subject to independent oversight, and responsibly managed to minimize the impact of conflicts of interest. Public health agencies and research laboratories alike need to open their records to the public. Investigators should document the veracity and provenance of data from which analyses are conducted and conclusions drawn, so that analyses are reproducible by independent experts.

Finally, in this time of unfortunate anti-Asian sentiment in some countries, we note that at the beginning of the pandemic, it was Chinese doctors, scientists, journalists, and citizens who shared with the world crucial information about the spread of the virus—often at great personal cost (8, 9). We should show the same determination in promoting a dispassionate science-based discourse on this difficult but important issue.
